# Assessing the Capability of Large Language Models for Navigation of the Australian Health Care System: Comparative Study

**DOI:** 10.2196/76203

**Published:** 2025-10-07

**Authors:** Joshua Simmich, Megan Heather Ross, Trevor Glen Russell

**Affiliations:** 1 RECOVER Injury Research Centre, Faculty of Health, Medicine and Behavioural Sciences, The University of Queensland, 288 Herston Rd, Queensland, Brisbane, 4029, Australia, +61 7 3365 5560; 2 STARS Education and Research Alliance, Surgical Treatment and Rehabilitation Service (STARS), University of Queensland and Metro North Health, Brisbane, Australia

**Keywords:** artificial intelligence, large language models, search engines, Australia, health services accessibility, questionnaires

## Abstract

**Background:**

Australians can face significant challenges in navigating the health care system, especially in rural and regional areas. Generative search tools, powered by large language models (LLMs), show promise in improving health information retrieval by generating direct answers. However, concerns remain regarding their accuracy and reliability when compared to traditional search engines in a health care context.

**Objective:**

This study aimed to compare the effectiveness of a generative artificial intelligence (AI) search (ie, Microsoft Copilot) versus a conventional search engine (Google Web Search) for navigating health care information.

**Methods:**

A total of 97 adults in Queensland, Australia, participated in a web-based survey, answering scenario-based health care navigation questions using either Microsoft Copilot or Google Web Search. Accuracy was assessed using binary correct or incorrect ratings, graded correctness (incorrect, partially correct, or correct), and numerical scores (0‐2 for service identification and 0‐6 for criteria). Participants also completed a Technology Rating Questionnaire (TRQ) to evaluate their experience with their assigned tool.

**Results:**

Participants assigned to Microsoft Copilot outperformed the Google Web Search group on 2 health care navigation tasks (identifying aged care application services and listing mobility allowance eligibility criteria), with no clear evidence of a difference in the remaining 6 tasks. On the TRQ, participants rated Google Web Search higher in willingness to adopt and perceived impact on quality of life, and lower in effort needed to learn. Both tools received similar ratings in perceived value, confidence, help required to use, and concerns about privacy.

**Conclusions:**

Generative AI tools can achieve comparable accuracy to traditional search engines for health care navigation tasks, though this did not translate into an improved user experience. Further evaluation is needed as AI technology improves and users become more familiar with its use.

## Introduction

Health care in Australia is recognized as both comprehensive and highly complex, making it challenging for many individuals to navigate [1,2]. Although the majority of Australians—around 86%—report navigating the system with relative ease, 14% find it difficult [1]. The challenge is particularly pronounced for health services in regional, rural, and remote contexts, where lack of awareness and the complexity of accessing care are cited as major barriers [3]. Moreover, rural Australians, who comprise roughly 28% of the population, experience higher rates of illness and lower per capita funding compared to their urban counterparts [4,5]. These disparities underscore the importance of effective tools and strategies that can simplify health care navigation, with implications for rural and regional populations.

To ensure that studies aimed at developing effective tools and strategies for simplifying health care navigation truly address the needs of communities, it is essential to involve health consumers directly in setting research priorities [6,7]. The research priority for this study was informed via a consumer engagement session held in Dalby, Queensland, a rural town approximately 200 kilometers west of Brisbane, on February 14, 2024. The roundtable brought together community members, regional health service providers, and researchers to discuss health care challenges specific to rural and remote populations. Participants highlighted significant barriers to accessing health care, particularly the difficulty of understanding what services are available and determining if they are eligible for them. Many described feeling uncertain about where to seek help, encountering confusing bureaucratic processes, and struggling to navigate both in-person and online health resources. Guided by this input, the present study focuses on health navigation challenges involving rural and remote health services in Queensland, Australia.

In recent decades, the internet has emerged as a primary source of health information for Australians of all ages, with a search engine being the most common starting point [8-11]. However, individuals searching for health information online often do not engage in thorough source comparison, instead extracting information from the search results page or a single website rather than critically assessing multiple authoritative sources [12]. This can lead to suboptimal decisions and increased barriers to timely, appropriate care, particularly in complex health care systems like that of Australia.

One technological innovation that may help address these challenges is the deployment of generative artificial intelligence (AI) in health information searches. Research indicates that users in Australia are already testing the waters with AI-based tools; for instance, nearly one in 10 Australians reported using ChatGPT to ask health questions in the first half of 2024 [13]. Large language model (LLM)–based systems can simplify health information so that it is more readable [14], potentially improving health information seeking. Emerging research suggests that clinicians generally prefer LLM-generated responses to common patient health queries, rating them as more accurate and comprehensive than the results provided by traditional search engines [15-19]. In addition, LLMs can be interfaced with search engines to create generative AI search tools (also known as conversational search), which promise to streamline information retrieval by automating the process of selecting search terms, filtering content, and providing a readable summary of information from multiple sources [20,21]. However, there are significant concerns about the reliability of LLMs. For instance, LLMs often “hallucinate,” generating information that appears plausible but is inaccurate [22,23]. In a health care context, where evidence-based information is critical, the consequences of these inaccuracies can be serious. Although early adopters may find these tools appealing, it is not clear whether generative AI search tools are as accurate as conventional search engines in helping Australians navigate the health care system.

The primary aim of the present study is to compare the accuracy of Australian users’ answers to health care navigation scenarios when using a generative AI search (Microsoft Copilot) versus using a conventional search engine (Google Web Search). The secondary aim is to compare the user experience of these 2 tools, in terms of factors such as perceived value, concerns about privacy, perceived effort to use, and willingness to adopt.

## Methods

Low/Negligible

### Participant Recruitment

We recruited participants using the web-based platform Prolific, who were then linked to Qualtrics to complete the survey. All Prolific users are required to be over 18 years of age. We only recruited those located in Australia and whose profile indicated they could speak English. To ensure all participants would have similar familiarity with state-based health policies and services, we screened out participants who resided in Australian states other than Queensland or had past or present employment in a health profession.

Participants were recruited between November 3, 2024, and January 8, 2025.

### Baseline Measures

Initial demographics were also collected, including age, gender (man, woman, nonbinary, or prefer not to disclose), and language proficiency. Additional data about participants’ eHealth literacy and computer skills and knowledge were collected using the eHealth Literacy Scale (eHEALS) [24]. The eHEALS includes 8 questions rated on a 5-point Likert scale, ranging from 1 (strongly disagree) to 5 (strongly agree), with a total possible score between 8 and 40.

In addition, a 4-item computer skills and knowledge questionnaire was used for the purpose of assessing use of computers, knowledge, skills with programs, and skills with computer applications [25]. Each item was rated on a 9-point rating scale, ranging from 1 (very low skill or knowledge) to 9 (very high skill or knowledge).

### Procedure

Participants were randomized to use either the generative AI search tool or Google Web Search. They were given simple instructions on how to access the site and interact with it; however, no guidance was provided on how best to formulate a prompt or search strategy.

Participants were tasked to complete 5 task-based scenarios, each presenting a brief vignette of a person, their location in rural or regional Queensland, their health condition, and the health service or health payment, subsidy, or scheme about which they were seeking information. Each scenario had 1 or 2 tasks respondents were asked to complete using their assigned tool (Table 1), for a total of 8 tasks. An example of a scenario (Scenario 3) and the 2 associated tasks is provided in Textbox 1 (all scenarios and tasks available in Multimedia Appendix 1). Although they were instructed to spend no more than five minutes per scenario, no time limits were enforced. Seven participants who did not provide a response to any of the 5 scenarios were removed from the sample.

**Table 1.** Task-based scenarios with corresponding outcome (and associated scoring criteria).

Scenario	Task	Outcome	Scoring
1	Locating telehealth physiotherapy clinics	Number of correctly identified services	0‐2
2	Locating nearby aged care providers	Number of correctly identified providers	0‐2
2	Naming aged care application service	Whether a service named is correct	Incorrect, correct
3	Listing mobility allowance eligibility criteria	Number of correctly identified criteria	0‐6
3	Providing mobility allowance helpline number	Whether a phone number provided is correct	Incorrect, correct
4	Locating nearby mental health center	Correctness of identified facility(name AND address)	Incorrect, partially correct, correct
5	Assessing patient travel subsidy eligibility	Correctness of subsidy identified	Incorrect, partially correct, correct
5	Providing travel subsidy agency contact details	Whether any contact details provided are correct	Incorrect, correct

Textbox 1. **An example of a scenario (Scenario 3) provided to participants, with the 2 tasks associated with this scenario.**

**Scenario 3**

Hamid sustained a spinal injury in a motor vehicle collision 9 months ago. He now uses a wheelchair permanently for mobility. He recently moved to live with family in Charters Towers and is looking for employment opportunities. However, he finds it difficult to pay for the cost of travel to look for work as he cannot drive and there is no public transport in the area. He has a membership for the Queensland Government's Taxi Subsidy Scheme (TSS), but this only pays half of his taxi fares. He recently learned of a funding program called Mobility Allowance.

*Task 1:*

What information about Hamid would you need to know to determine if he is eligible for this program?

*Task 2:*

Hamid wants to talk to somebody over the telephone about getting mobility allowance, but struggles with speaking English. What phone number(s) should he call?

### Model Selection

**Figure 1.**
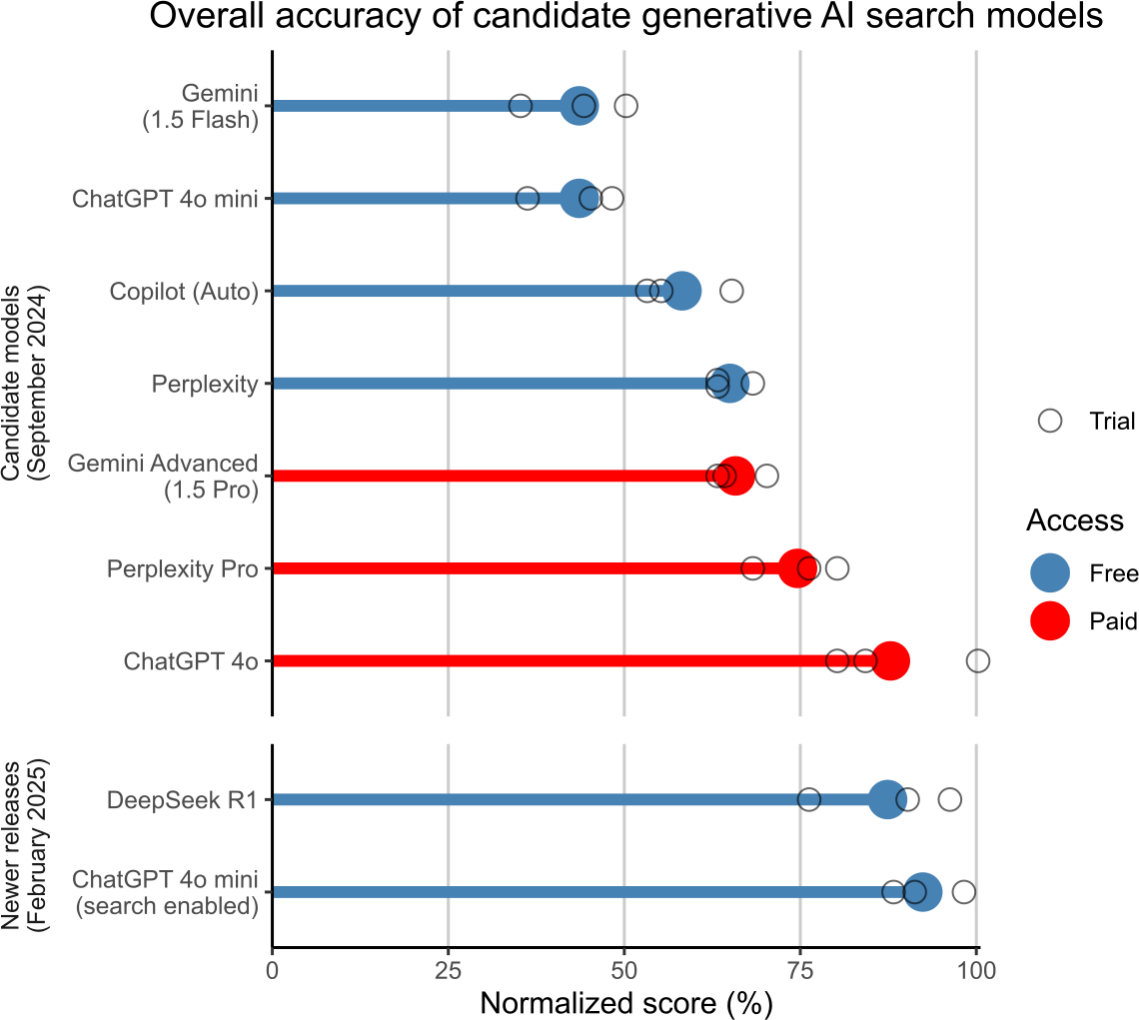
Chart of mean accuracy of the candidate free-access and subscription-only generative AI search tools across 3 trials. Results for each trial indicated by empty circles.

To determine which generative AI search tool would be more appropriate for the current study, the accuracy of various free-access and subscription-only generative AI search tools was assessed. In September 2024, using the same prompts as detailed above, each answer engine was prompted 3 times. Each trial was conducted in an independent session (ie, a new chat was started each time), with any ’memory’ features across chats disabled where applicable. The responses were scored by one author (JS), using the same scoring metric as the primary outcome measure, and scores were discussed with the research team. An equal-weighted total average score for each model was calculated by assigning numeric scores to all binary or ordinal scores (eg, Incorrect=0, Partially correct=0.5, Correct=1), weighting each of the 5 scenarios equally. By this metric, all subscription-only generative AI search tools available at the time were substantially more accurate than the free-access versions available at the time (Figure 1). However, it was not feasible to supply all survey participants with access to a paid subscription. Of the free-access tools available at the time, Microsoft Copilot (formally Bing Chat) was chosen due to not requiring user accounts to be created for access, which streamlined the survey process and decreased the burden on participants. For additional context, newer free models released after the study began were tested in February 2025 and are also shown in Figure 1.

### Outcome Measures

#### Primary Outcome Measure

The primary outcome variable was the accuracy of the response, based on whether correct information was retrieved. One author (JS) developed a scheme to score each response, which was reviewed and refined based on feedback from a second author (MHR). Two authors (JS, MHR), both registered physiotherapists in Queensland, scored each response based on these criteria and resolved any disagreements through discussion. Scoring was blinded to the search tool used to generate the answers. Points were awarded based on the presence of correct information (eg, services or payments that would address the client’s needs, correctly identified eligibility criteria, or correct contact details). Scores were not deducted for the presence of incorrect information in the responses, nor was the score deducted for providing more information than asked (eg, if asked for 2 potential services and giving 3).

#### Secondary Outcome Measure

Responses to a Technology Rating Questionnaire (TRQ) [25] were collected as secondary outcomes. Participants were asked to respond to 7 items on a 1-9 numerical rating scale (anchored by 1 as none or not at all and 9 as a lot or extremely) about the technology, including their:

- Willingness to adopt
- Confidence in learning
- Help needed to learn
- Perceived effort to learn
- Perceived value
- Privacy concerns
- Quality of life impact

### Data Analysis

Data were analyzed using R software (version 4.4.1; R Core Team). To compare the 2 groups (Microsoft Copilot and Google Web Search) for the primary outcome of how well responses matched the pre-set criteria, appropriate statistical methods were selected for each question. For questions assessed on a continuous or interval scale (eg, number of correct answers), the mean difference between scores was presented alongside boot-strapped 95% CIs generated with the “boot” package, using the bias-corrected and accelerated (BCa) method with 5000 iterations. For questions with binary answers (ie, marked as either correct or incorrect), the difference in proportions of correct responses between groups was presented alongside the 95% CIs calculated with the “DescTools” package using the Agresti-Coull method. For questions with ordinal answers (eg, marked as Incorrect, Partially Correct, or Correct), ordinal regression was performed using cumulative link models with the “ordinal” package, with a logit link function. The proportional odds assumption was verified using Brant tests with the package “brant.” The secondary outcome on the technology rating questionnaire was analyzed using the same bootstrapping method as the interval scale data for the primary outcome.

### Ethical Considerations

This study was approved by The University of Queensland Faculty of Health and Behavioural Sciences Low and Negligible Risk Panel (2024/HE001343). Participants provided electronic informed consent after reviewing a participant information sheet. Recruitment occurred via a research panel (Prolific), and—given the anonymous design—data provided before withdrawal could not be removed. Participants were compensated for their time through Prolific, receiving an average of £5.50 (approximately US$6.90). We maintained participants’ privacy and confidentiality by collecting only nonidentifiable data, which were stored on The University of Queensland Research Data Management System.

## Results

### Sample Characteristics

After receiving 672 submissions from Prolific, the final sample consisted of 97 participants. Most participants were from metropolitan areas (72/97, 74%). Mean age was 36 (SD 14) years with an age range from 18 to 83 years. Gender distribution included 49% (48/97) identifying as men, 42% (41/97) as women, and 3% (3/97) as nonbinary. Only 6% (6/97) of the sample reported being of Aboriginal or Torres Strait origin. See Table 2 for full details.

The mean self-reported eHEALS of participants (mean sum score) was 27 (SD 4; min 12, max 35) out of 40, indicating most participants were confident using the internet for health information. The average rating for computer and internet skills and knowledge was around 7.5-8 (of a range of 1-9), indicating participants were very confident with the use of computers and the internet.

**Table 2.** Participant sociodemographic and health characteristics, computer and Internet skills, and eHealth literacy scores.

Characteristic	Google Web Search (n=50)	Microsoft Copilot (n=47)	Overall (n=97)
What is your age (in years)? mean (SD)	37 (14)	35 (15)	36 (14)
With which gender do you most identify? n (%)			
Man	29 (58)	19 (40)	48 (49)
Woman	18 (36)	23 (49)	41 (42)
Nonbinary	1 (2)	2 (4)	3 (3)
Are you of Aboriginal or Torres Strait origin? n (%)			
No	45 (90)	41 (87)	86 (89)
Yes, Aboriginal	3 (6)	3 (6)	6 (6)
What is your highest level of completed education? n (%)			
High school (secondary school)	5 (10)	8 (17)	13 (13)
Certificate I-IV (including trade certificate)	6 (12)	6 (13)	12 (12)
Diploma (or associate degree)	4 (8)	2 (4)	6 (6)
Bachelor degree (including Bachelor Honours degrees and graduate diploma or certificate)	21 (42)	21 (45)	42 (43)
Masters degree (coursework or research)	10 (20)	3 (6)	13 (13)
Doctoral degree (eg, PhD)	1 (2)	4 (9)	5 (5)
Prefer not to say	1 (2)	0 (0)	1 (1)
What is your current employment status? n (%)			
Employed (full time)	22 (44)	21 (45)	43 (44)
Employed part time (or casual employment)	12 (24)	12 (26)	24 (25)
Student and employed	1 (2)	4 (9)	5 (5)
Student, not employed	2 (4)	3 (7)	5 (5)
Retired	3 (6)	0 (0)	3 (3)
Unemployed, looking for work	5 (10)	3 (6)	8 (8)
Person with disability and unable to work	2 (4)	0 (0)	2 (2)
Student, with disability and unable to work	0 (0)	1 (2)	1 (1)
Self-employed	1 (2)	0 (0)	1 (1)
Geographic remoteness (Modified Monash Model [MMM] 2019), n (%)			
MMM 1: Metropolitan areas in major cities	39 (78)	33 (70)	72 (74)
MMM 2: Regional centers (populations >50,000)	2 (4)	6 (13)	8 (8)
MMM 3: large rural towns (15,000–50,000)	1 (2)	1 (2)	2 (2)
MMM 4: medium rural towns (5000–15,000)	1 (2)	1 (2)	2 (2)
MMM 5: small rural towns (<5000)	4 (8)	2 (4)	6 (6)
Do you have a chronic health condition? n (%)			
No	32 (64)	31 (66)	63 (65)
Prefer not to say	0 (0)	2 (4)	2 (2)
Yes, 1 chronic health condition	12 (24)	7 (15)	19 (20)
Yes, several chronic health conditions	4 (8)	3 (6)	7 (7)
Do you use a language other than English at home? n (%)			
No, English only	45 (90)	36 (89)	81 (92)
Yes, use another language at home	3 (6)	8 (17)	11 (11)
How well do you speak English? n (%)			
Very well	47 (94)	42 (89)	89 (92)
Well	1 (2)	2 (4)	3 (3)
Not very well	0 (0)	0 (0)	0 (0)
Not at all	0 (0)	0 (0)	0 (0)
eHealth Literacy Score (eHEALS), mean (SD)	27 (4)	27 (4)	27 (4)
Computer and Internet skills and knowledge (1 to 9 scale), mean (SD)			
Basic computer skill	8.1 (1.1)	8.3 (1.0)	8.2 (1.0)
Internet and email skill or knowledge	8.0 (1.0)	8.1 (1.0)	8.1 (1.0)
Computer programs knowledge	7.6 (1.3)	7.6 (1.3)	7.6 (1.3)
Computer applications knowledge	7.7 (1.3)	7.8 (1.1)	7.8 (1.2)

### Primary Outcome

Participants in the present study using Microsoft Copilot achieved an overall accuracy averaging 68.2% (equally weighted across all tasks), while those using Google Web Search averaged 65.9%. Median length of participants’ written responses across all tasks in the Microsoft Copilot was 158 characters (IQR 37-373; Min=7, Max=1558), whereas in the Google Web Search group median response length was 65 characters (IQR 16-104; Min=3, Max=1492), a median difference of 93 characters (95% CI 53-138).

**Figure 2.**
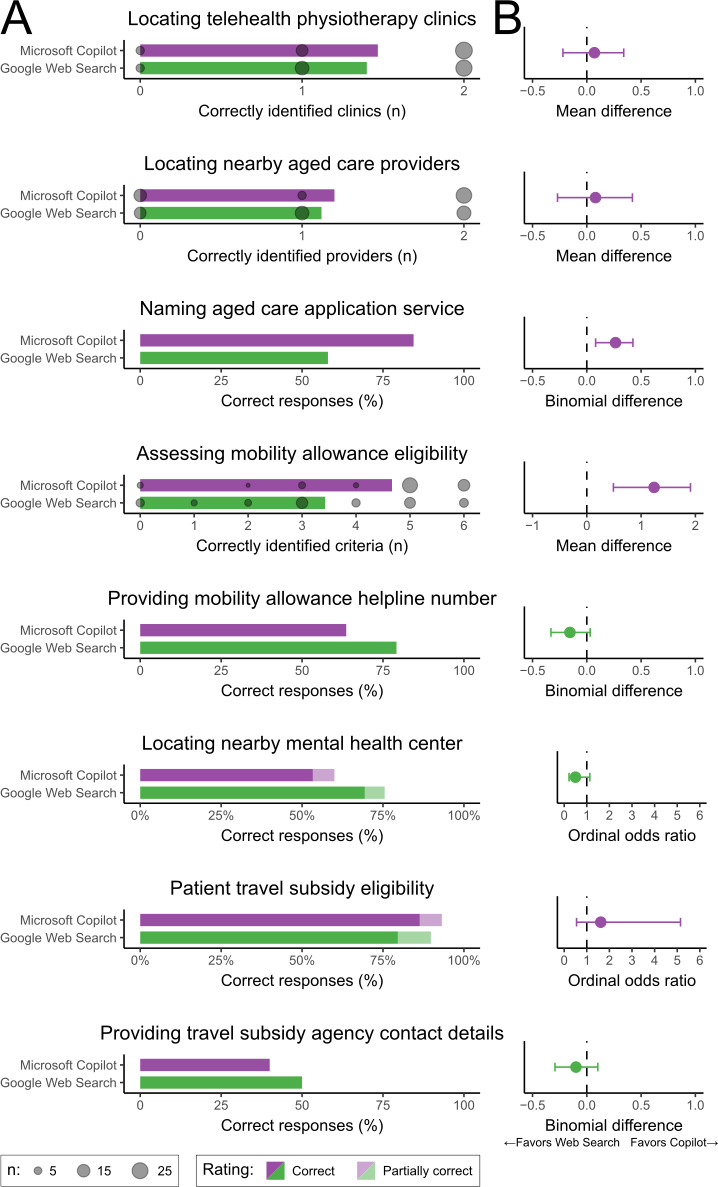
Accuracy of responses to healthcare navigation tasks using Microsoft Copilot versus Google Web Search. (A) Bar charts indicating response accuracy ratings across eight navigation tasks. (**B**) Between-group comparisons, with associated 95% CI.

In the Scenario 1 task (locating telehealth physiotherapy clinics), respondents randomly assigned to Microsoft Copilot correctly located an average of 1.47 telehealth services, just 0.07 (95% CI −0.22 to 0.34) more than the 1.40 services found by those assigned Google Web Search (Figure 2).

In the first Scenario 2 task (locating nearby aged care providers), respondents assigned to Microsoft Copilot identified an average of 1.20 services, only 0.08 (95% CI −0.27 to 0.42) services more than the 1.12 services identified by those assigned to Google Web Search. In the second Scenario 2 task (naming aged care application service), 84% (38/45) of respondents in the Microsoft Copilot group identified the correct government service compared with 58% (29/50) who did so in the Google Web Search group. This indicates Microsoft Copilot increased the proportion of correct responses for this question by 0.26 (95% CI 0.08-0.42) over Google Web Search (Figure 2).

In the first Scenario 3 task (listing mobility allowance eligibility criteria), Microsoft Copilot respondents correctly identified an average of 4.67 eligibility criteria compared to 3.43 criteria in the Google Web Search group (Figure 2). This represents a clear advantage in favor of Microsoft Copilot, with a mean difference of 1.24 (95%CI 0.49-1.91). In the second Scenario 3 task (providing mobility allowance helpline number), just 28 of the 44 Microsoft Copilot group listed a correct phone number for the translation service, which was a lower proportion than the 38 of 48 respondents assigned Google Web Search (Figure 2). This is a mean difference in proportions of −0.16 (95% CI −0.33 to 0.03).

For the Scenario 4 task (locating a nearby mental health center), 53% (24/45) of respondents in the Microsoft Copilot group were completely correct and 3 of the 45 were partially correct. In contrast, 69% (34/49 respondents) in the Google Web Search group were completely correct and 3 of 49 were partially correct (Figure 2). Estimated odds ratio from the ordinal logistic regression analysis of 0.5 (95% CI 0.2-1.1) suggests that assignment to Microsoft Copilot may substantially reduce the odds of achieving a higher score relative to Google Web Search. However, the wide confidence interval indicates imprecision, with possible effects ranging from a large reduction through no difference to even a modest increase.

In the first Scenario 5 task (assessing patient travel subsidy eligibility), 86% (38/44 respondents) in the Microsoft Copilot group answered completely correctly, with an additional 3 (of the 44) providing partially correct answers (Figure 2). For the same question, 80% (39/49 respondents) in the Google Web Search group were completely correct, and 5 (of the 49) offered partially correct responses. Based on ordinal logistic regression, participants assigned to Microsoft Copilot had approximately 1.6 times the odds of achieving a more accurate answer compared to those in the Google Web Search group (odds ratio [OR] 1.6, 95% CI 0.5-5.2). However, the wide confidence interval ranges from a reduction in odds to a substantial increase. In the second Scenario 5 task (providing travel subsidy agency contact details), just 40% (18/45) of respondents in the Microsoft Copilot group listed correct contact details for the government agency, whereas exactly half of those in the Google Web Search group (24 of 48) did so (Figure 2). This is a difference in proportions of −0.1 (95% CI −0.29 to 0.10).

### Secondary Outcome (Technology Rating Questionnaire)

**Figure 3.**
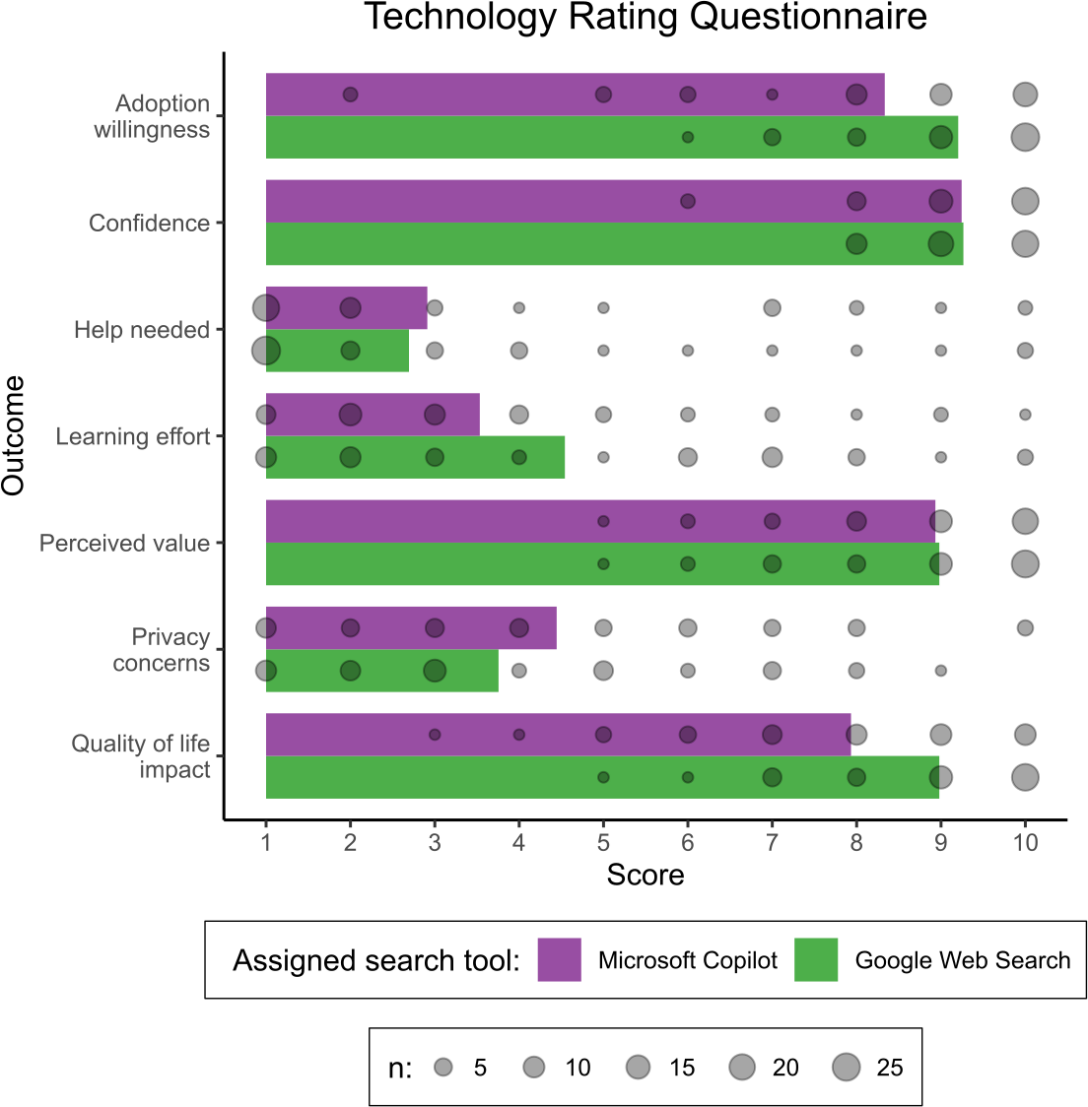
Combined bar chart and bubble chart showing mean score (bars) and counts (bubble area) for score ratings on the technology rating questionnaire for each assigned tool.

On the 0‐10 TRQ subquestions (Figure 3), participants on average rated Microsoft Copilot as 0.87 points lower for willingness to adopt (95% CI −1.65 to −0.28) and 1.05 points lower for impact on quality of life (95% CI −1.69,−0.42). These 95% CIs are compatible with meaningful differences favoring Google Web Search, though the precise effect sizes remain uncertain.

For perceived value (mean difference −0.05, 95% CI −0.57 to 0.49), help needed (mean difference 0.22, 95% CI −0.89 to 1.33), and confidence (mean difference −0.02, 95% CI −0.43 to 0.32), the confidence intervals are all compatible with small effects in either direction or essentially no difference between the 2 tools.

Privacy concerns were 0.69 points higher for Microsoft Copilot (95% CI −0.32 to 1.67), though the confidence interval includes the possibility of no difference or even slightly greater concerns about Google Web Search.

Finally, participants reported that Google Web Search required 1.01 points less effort to learn than Microsoft Copilot (95% CI −1.69 to −0.42), consistent with Google Web Search being perceived as somewhat easier to learn than Microsoft Copilot.

## Discussion

### Summary of Findings

The present study compares the occurrence of correct information in responses to health navigation queries from users assigned to use either a generative AI search tool (powered by an LLM augmented with the retrieval of live web data) or a traditional search engine. Our primary results found those assigned to Microsoft Copilot outperformed the Google Web Search group for 2 tasks: naming the service to apply for an aged care assessment and assessing mobility allowance eligibility. There was no clear evidence of differences between the tools for the remaining 6 tasks. This suggests that while this generative AI search tool might be superior to a search engine in some specific instances, its overall advantage in terms of accuracy in health navigation tasks is not guaranteed.

We acknowledge that our selection of Microsoft Copilot as the generative AI search tool may limit generalizability to other current and future generative AI search tools. Microsoft Copilot was selected because it offered free access without the need for participants to create an account. However, the field of AI is evolving rapidly, with many newer and more advanced search-enabled models being released by OpenAI, DeepSeek, Anthropic, and others. Indeed, our initial testing of various models (Figure 1) indicates that many other generative AI search tools were substantially more accurate than Microsoft Copilot on the health navigation tasks used in the present study. Therefore, if participants had been assigned a more advanced generative AI search tool, it is possible they would have achieved higher accuracy compared to Google Web Search.

The growing integration of generative AI into conventional web search has blurred the lines between traditional search engines and AI-based search tools, potentially influencing the outcomes of the present study. In October 2024, just one month before recruitment commenced for the present study, Google rolled out AI Overviews across Australia, a generative AI feature that provides users with concise, AI-generated summaries within search results [26]. However, these summaries would (at the time) only appear when users were logged in using their Google account and participants assigned to Google Web Search were instructed to open a private (or incognito) window to ensure they were not logged in. Despite this, participants who did not follow these instructions could potentially have encountered these AI-generated summaries. Participants were responding to a web-based survey, and therefore, it was not possible to track or record their exact search queries or which websites were visited. As generative AI becomes more embedded in everyday search experiences, traditional web searches could potentially become less common. This may make studying search engines in isolation less practically relevant and increase the need for ongoing research into the accuracy and reliability of generative AI search.

It is noteworthy that the Microsoft Copilot group achieved an overall accuracy of 68.2%, compared with 58.2% observed during preliminary testing conducted 2 months earlier, where scenarios were simply copy-pasted into Copilot 3 times. The higher accuracy observed might suggest that participants visited source websites to verify the information in the AI-generated summaries. It could also result from participants using more effective prompting strategies (than using the question verbatim), asking follow-up questions, or retrying prompts when initial responses were clearly incorrect. However, this is an incidental observation rather than a study objective. We cannot exclude sampling bias, given our preliminary estimate was based on just 3 attempts. In addition, improvements in Copilot’s underlying AI model or updates to the accuracy of content available on the web between preliminary testing and study commencement could also have contributed to this difference.

Participants in our study skewed toward higher formal education and were generally quite confident in their ability to find health information on the internet. This may limit generalizability, as individuals with lower educational levels have lower self-rated ability to evaluate online health information and report lower overall trust in such information, compared to their more educated counterparts [27]. Furthermore, some evidence suggests that generative AI tools tend to offer greater benefits for nonexperts [28], though other evidence suggests no advantage in favor of ChatGPT (without web search capability) among lower-education users with lower proficiency using Google Web Search [29]. It is possible that most participants in our study were able to effectively complete the task using either tool, potentially decreasing any between-tool differences. We did not design or power the study to test differences by education or eHealth literacy, so any differential effects remain a question for future work.

Health care navigation is a multifaceted process, depending not only on access to accurate information, but also consideration of the affordability of the health service, flexibility of service options, inclusivity of the clinical environment, and alignment with consumers’ personal beliefs and knowledge [30]. Given this complexity, the scenario-based tasks used in this study likely do not fully capture the nuances of real-world health care navigation. Participants were presented with contrived scenarios in which they had no personal or emotional investment, only a single attempt to find the correct answer within a brief 5-minute window, with no opportunity to reassess or refine their responses, and scored on an artificial scoring metric. The scenarios were also deliberately designed to be challenging yet solvable, specifically to enable a meaningful comparison between the 2 tools, rather than to represent the most common real-world health navigation scenarios. Therefore, performance on these scenarios cannot be used to draw definitive conclusions about real-world health navigation ability. Future research exploring generative AI in authentic health care contexts should better account for these nuances and thus more accurately represent how individuals engage in health decision-making.

Secondary outcome results indicate that the willingness to adopt Google Web Search was higher than Microsoft Copilot. This contrasts with a previous study, which found that despite greater trust in an LLM (OpenAI’s ChatGPT) for health information, participants rated intention to use the LLM similarly to Google Web Search [31]. This difference may be due to familiarity with the specific LLM, as ChatGPT is more widely used than Copilot for health-related queries [32], potentially leading to greater willingness to use. More broadly, users’ trust in health information and chatbots is shaped by factors such as usability, perceived risk, and credibility [33,34], all of which could differ between ChatGPT and Copilot. Furthermore, the difference in tasks between seeking health information and navigating health care services might influence users’ trust and adoption decisions, especially if there is a perception that LLM-based systems can struggle with more context-dependent or location-specific queries.

The present study found no clear evidence of a difference in perceived effort to use or help required between the generative AI search and conventional search, in concordance with existing research comparing ease of use of LLMs and search engines for health queries [32]. Contrasting our findings, a recent study [35] reported lower cognitive load among students researching socio-scientific issues about sunscreen with ChatGPT compared to Google. Participants may have greater concerns about the reliability and accuracy of AI-generated health information than other types of information, potentially limiting the advantages of AI-driven tools in a health context. In addition, it has been reported that users tend to prefer traditional search engines for fact-based information retrieval, turning to LLMs primarily when they need more personalized or lay-language explanations [36]. Because the health navigation tasks in this study were relatively fact-based, the strengths of a generative AI search tool may not have been prominent.

### Strengths and Limitations

A key strength of this study is that it closely observed how real users interact with 2 distinct search tools, rather than merely comparing the static accuracy of the output of each tool. By allowing participants to formulate queries and responses freely, a more realistic picture of AI-assisted health care navigation was obtained. In addition, focusing on generative AI search—rather than an LLM without the capability to augment responses with information from a web search—better reflects the tools now emerging in consumer products. Our relatively large sample size of approximately 100 participants also provides a solid basis for analyzing differences between the 2 groups.

Despite these strengths, an important methodological limitation lies in our scoring approach, which looked only at the presence of correct information in responses and did not account for the presence of incorrect information. Not penalizing incorrect information means our scores may overestimate the accuracy of responses, since in real-world health navigation contexts, misinformation can delay care or impose additional burden on users. Although when compared to other health contexts (such as clinical advice), such risks may be somewhat less impactful for health navigation, as users may receive assistance when contacting incorrect government services, these risks may still be important for urgent health navigation tasks. Consequently, longer responses with multiple possible answers may have received inflated accuracy scores due to an increased chance of including correct items. This may have favored the Microsoft Copilot group, perhaps because they could easily copy the generated AI response, responded with much longer answers to the scenarios than the Google Web Search group. Similarly, our scoring metric may not have been able to capture the practical value of receiving partially helpful information, such as being directed to call a general help line (marked as incorrect in the scoring metric) that could have referred callers to the more appropriate service (which the metric marked as correct). We therefore highlight that our metric should be interpreted as measuring whether users can obtain correct information with these search tools, rather than whether they do so without adding incorrect information.

In addition to the scoring issues, several other methodological limitations stem from participant characteristics and study context. Participants in our sample were predominantly living in metropolitan areas and may have had limited direct experience with rural health care services, which may have affected how they interpreted the navigation tasks of the present study. Furthermore, we did not explore the extent to which participants were familiar with their assigned tool, and familiarity (or lack thereof) could have affected both the accuracy of the responses and how participants rated the technology. Overall, these methodological constraints mean that our findings should be interpreted cautiously, especially when extending them beyond the specific population, tasks, or tools studied here.

Future research should explore these findings further by conducting a follow-up study focused exclusively on rural participants to determine whether the observed patterns persist in different geographic contexts. In addition, future studies should prioritize methods that can more precisely capture user behavior, such as incorporating screen-tracking software or controlled environments, to yield more definitive insights. Another critical avenue for investigation is how users engage with lengthy LLM-generated responses, particularly how they prioritize or dismiss certain portions when seeking health-related information. Understanding this selection process could inform strategies to improve AI-generated content for critical decision-making. Finally, longitudinal research could help clarify whether repeated exposure to AI-generated content enhances users’ ability to critically assess multiple answers or, conversely, reinforces reliance on AI as an authoritative source.

### Conclusions

Although Microsoft Copilot demonstrated improved accuracy over Google Web Search on 2 of the tested scenarios, for all others, the accuracy estimates were compatible with no difference between the 2 tools. Participants also reported lower willingness to adopt Copilot, as well as higher concerns around privacy and ease of learning. The results of this study underscore how generative AI tools still face hurdles in accuracy and user acceptance. Future work should investigate whether newer and more capable generative AI tools can consistently outperform conventional search engines across a broader range of real-world health navigation tasks.

## Supplementary material

10.2196/76203 Multimedia Appendix 1 Text of all scenarios and associated tasks.

## Abbreviations

AI: artificial intelligence
eHEALS: eHealth Literacy Scale
LLM: large language model
TRQ: Technology Rating Questionnaire
